# Performance and Microstructure of Grouting Materials Made from Shield Muck

**DOI:** 10.3390/ma17164074

**Published:** 2024-08-16

**Authors:** Zhenxu Wu, Chaoliang Ye, Fengxu Cao

**Affiliations:** School of Civil Engineering, Shijiazhuang Tiedao University, Shijiazhuang 050043, China; azwuzx@163.com (Z.W.); caofxu@stdu.edu.cn (F.C.)

**Keywords:** shield tunnel, synchronous grouting, muck recycling, orthogonal test, microstructure

## Abstract

In response to the environmental pollution caused by transportation and accumulation of large-scale shield muck, the on-site reutilization of shield muck is an effective approach. This study explored the feasibility of silty clay muck to prepare muck grout. Through orthogonal experiments, the effects of cement, fly ash, shield muck, admixture, and the water–solid ratio on the fresh properties and mechanical properties of muck grout were studied. The performance prediction model was established Additionally, the intrinsic relationships between the compressive strength and microstructure of shield muck grouting materials were explored through multi-technology microstructural characterization. The results indicate that the content of muck and the water–solid ratio have a greater significant influence on the bleeding ratio, flowability, setting time, and volume shrinkage rate of muck grout compared to other factors. Cement has a greater significant influence on the compressive strength of muck grout than other factors. An optimal mix proportion (12% for cement, 18% for fly ash, 50% for muck, 0.465 for water–solid ratio, 19.5% for river sand, and 0.5% for bentonite) can produce grouting materials that meet performance requirements. The filling effect of cementitious substances and the particle agglomeration effect reduce the internal pores of grouting materials, improving their internal structure and significantly enhancing their compressive strength. Utilizing shield muck as a raw material for shield synchronous grouting is feasible.

## 1. Introduction

With the rapid development of underground space, shield tunneling has been widely used in construction projects such as rail transportation, municipal highways, and urban comprehensive pipe corridors due to its advantages of high efficiency, safety, and mechanization [1,2]. However, the shield construction process generates a massive amount of muck. According to incomplete statistics, the annual total volume of shield muck generated by shield tunnels in China has exceeded 225 million cubic meters, with disposal costs estimated to reach CNY 58.2 billion [3]. Shield muck has characteristics such as small particle size, high water content, and poor stability, making it difficult to be completely recycled using traditional construction waste methods. Currently, shield muck is still treated by landfill and stacking [4,5], which not only occupies land resources and increases costs but also causes severe environmental pollution such as dust, noise, and spillage during transportation. Although scholars have used shield muck to produce sintered bricks [6], high-belite cementitious materials [7], recycled aggregates [8], and subgrade materials [9], the actual utilization rate of shield muck in China is less than 1% [10]. The disposal and utilization of large volumes of shield muck have become a major challenge hindering the safe and efficient construction of shield tunnels.

In recent years, the use of shield muck to prepare grouting materials has become a research hotspot. This approach can effectively prevent environmental pollution and disasters such as landslides caused by shield muck accumulation, while significantly reducing carbon emissions during the extraction and processing of grouting raw materials [11]. Vinai et al. explored the changes in slump and strength of shield muck under different water content and foam content through laboratory tests [12]. Zhou and Zhang successfully applied shield muck treated by the slurry system in backfill grouting [13,14]. Wang et al. found that the slurry prepared from sandy muck with appropriate material proportions can meet the performance requirements of synchronous grouting and validated the effectiveness of muck grout through monitored values such as surface settlement and segment uplift [15,16,17]. Although there have been reports on the application of shield muck in synchronous grouting, most focus on sandy soil or silty clay in slurry shields, with less research on the application of silty clay in earth pressure balance shields (the muck in slurry shields differs from that in earth pressure shields due to differences in properties). Given the different particle compositions of silty clay and sandy soil, the suitability of grouting materials must differ [18,19]. Some studies have shown that partially replacing river sand with silty clay reduces the flowability of backfill grouting and extends setting time [20,21], while high water content can lead to increased bleeding and reduced strength [22]. Therefore, further research is needed to prepare grouting materials from silty clay muck that meet the performance requirements of synchronous grouting.

This paper takes the silty clay muck from an excavation tunneling section of the Yinhan Jiwei Project as an example to explore the feasibility of using silty clay muck as a synchronous grouting raw material. Through orthogonal experiments, the fresh and mechanical properties of shield muck grouting materials (SMGM) with different material proportions were tested. The impact of material composition on their performance was analyzed, and a performance prediction model was established. Based on the performance requirements, suitable mix proportions for grouting materials were recommended. Additionally, the solidification mechanism of the SMGM was described at the microscopic level using various techniques such as XRD, SEM, and CT. This study provides a reference for the application of shield muck in synchronous grouting. It is of great significance for reducing costs, improving construction efficiency, and minimizing the environmental impact of synchronous grouting materials.

## 2. Experimental Scheme

### 2.1. Materials

The soil samples were obtained from a shield construction site in Xianyang City, Shanxi province of China. It was bagged and transported to the laboratory for natural air drying and was mechanically sieved through a 2 mm sieve mesh. According to GB50123-2019 [23], the relevant physical properties were tested. Its average values of dry density, plastic limit, liquid limit, and plasticity index are 1.55 g/cm^3^, 18.9%, 33.8%, and 14.9, respectively. The soil layer in this area is loess, primarily composed of silt and clay, characterized by high porosity and permeability.

**Figure 1:**
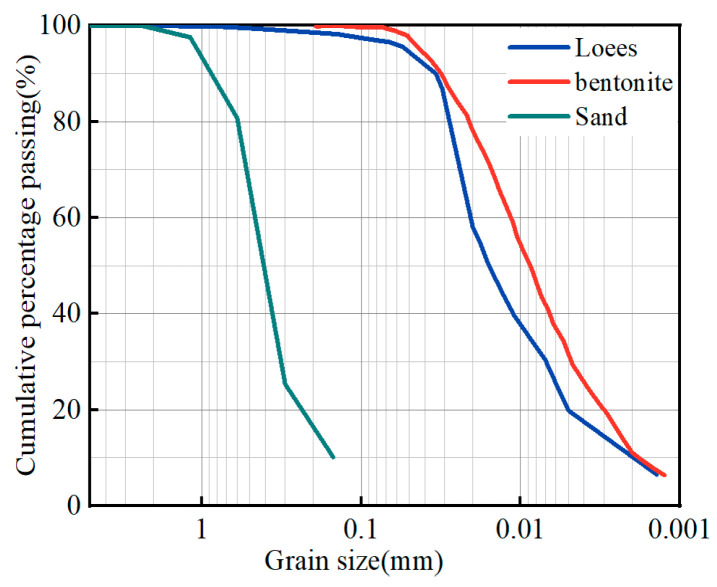
The particle size distribution of sand, bentonite, and loess.

**Figure 2:**
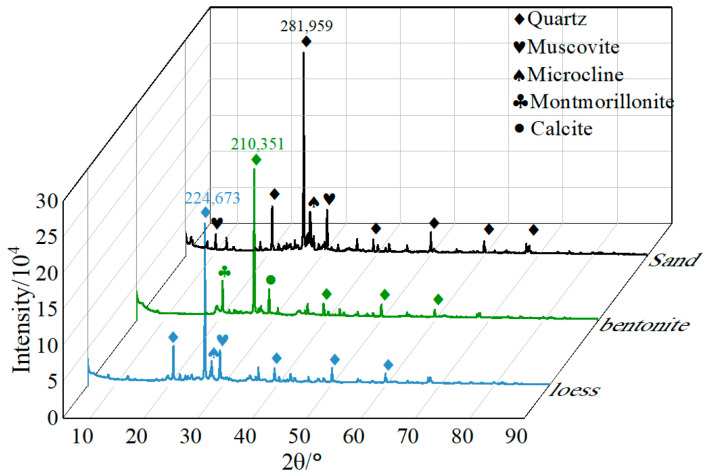
The XRD of sand, bentonite, and loess.

The cementitious materials were P.O 32.5 ordinary Portland cement and fly ash produced in Shijiazhuang City, Hebei province of China. The bentonite was calcium-based bentonite. According to the data provided by the manufacturer, the chemical composition of cement and fly ash is shown in Table 1, and the detailed parameters of bentonite are listed in Table 2. The fine aggregate was natural river sand, with an apparent density of 2600 kg/m^3^ and a fineness modulus of 2.79. The particle size distributions of loess, river sand, and bentonite are shown in Figure 1. Loess and bentonite have a high clay content. The mineral composition was determined using XRD testing, and the results are presented in Figure 2. The main minerals of river sand, bentonite, and loess are quartz, which is stable in nature and generally does not react with other substances [24]. Besides quartz, loess and river sand contain stable minerals such as feldspar, calcite, and muscovite. Therefore, from the perspective of mineral composition, the muck can be used as a raw material for backfill grouting. The admixture was a polycarboxylate superplasticizer with a water reduction rate of 25% and sodium tetraborate, which has a retarding effect. Based on preliminary tests, the composition of the admixture used in this study was superplasticizer:tetraborate = 1:1.

### 2.2. Experimental Design

Based on relevant studies and preliminary tests [25,26,27], an orthogonal experiment was conducted to design the mix proportions for SMGM [28]. The orthogonal experiment factors included the content of cement, fly ash, shield muck, admixture, and the water–solid ratio, with each factor set at four levels as shown in Table 3. The amount of admixture was expressed as a percentage of the cementitious materials. The L16(4^5^) orthogonal design was used for the experiment. Relevant tests were carried out on 16 groups of SMGM, and the test results are shown in Table 4.

### 2.3. Grout Preparation

The preparation of the SMGM was conducted as follows. Initially, the necessary dry materials (cement, fly ash, muck, sand, and bentonite) were added to the mixing pot and stirred for 3 min. Subsequently, water mixed with an admixture was combined with the dry ingredients. Stirring was continued for an additional 3 min to produce the SMGM. It was important to note that the moisture content of the shield muck and river sand was considered when calculating the water–solid ratio.

### 2.4. Test Methods

**Figure 3:**
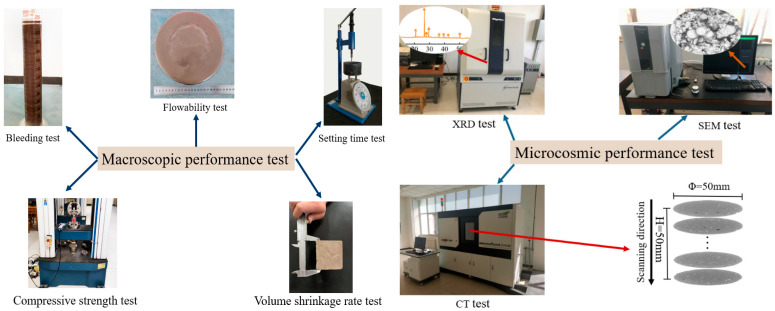
Performance test.

This paper tests the performance of the SMGM from three aspects: fresh properties, mechanical properties, and microstructure properties, as shown in Figure 3.

#### 2.4.1. Fresh Property Tests

The fresh properties of the SMGM include bleeding ratio, setting time, and flowability. The bleeding ratio of the SMGM was tested according to T/CECS563-2018 [29]. The fresh slurry was poured into a graduated cylinder of 250 mL and sealed with plastic film. After allowing the slurry to stand for 1 min, the initial volume of the slurry was recorded. The bleeding ratio was calculated as the ratio of the volume of water separated from the slurry after 3 h to the initial volume of the slurry. The setting time of the SMGM was tested according to the JGJ/T70-2009 [30]. The setting time was determined by the penetration resistance method using a ZKS-100 setting time tester. The container holding the slurry was placed on the pressure gauge dial. The pressure gauge needle was set to zero by adjusting the nut. The penetration needle was then brought into contact with the surface of the slurry and uniformly pressed into the slurry to a depth of 25 mm within 10 s. The gauge reading was recorded each time the needle penetrated. The setting time of the slurry was determined when the penetration resistance reached 15 N. The flowability of the SMGM was tested according to the GB/T 50448-2008 [31]. The test apparatus was a truncated cone mold with a 70 mm top diameter, 100 mm bottom diameter, and 60 mm height. The mold was placed on a 50 × 50 cm glass plate. First, fresh slurry was poured into the mold. Then, the mold was lifted vertically to allow the slurry to flow freely for 30 s. The average of the maximum diffusion diameter and its perpendicular diameter is taken as the flowability of the slurry. In addition, according to the actual engineering application, it takes 0–3 h from the completion of fresh grout to be injected into the shield tail gap. Therefore, the flowability of the SMGM was tested every 1 h after the fresh grout was complete. It was stirred again for 3 min before each test.

#### 2.4.2. Mechanical Property Tests

The mechanical properties of the SMGM include volume shrinkage rate and compressive strength. The volume shrinkage rate was calculated as the ratio of the difference between the actual volume of the sample and the standard volume of the mold to the standard volume of the mold [32]. The compressive strength of the SMGM was tested according to JGJ/T70-2009 [30] by using the YHS-229WJ electronic universal testing machine. The sample size was 70.7 mm × 70.7 mm × 70.7 mm. The sample was placed at the center of the pressure platform, ensuring the upper platen was in contact with the sample surface. The loading rate was adjusted to 1 mm/min, and loading was continued until the sample failed. The compressive strength of the sample was then recorded. The compressive strength of the SMGM was tested after 1 d, 3 d, 7 d, and 28 d of curing. Three parallel samples were used, and the average value was taken as the compressive strength.

#### 2.4.3. Microstructure Property Tests

The microstructure of the SMGM hardened for 28 d was characterized by using XRD, SEM, and X-CT. The mineral composition of the SMGM was examined by using an SmartLab(9)kW XG X-ray diffraction instrument (Rigaku Corporation, Tokyo, Japan). Samples were placed in an oven at 105 °C to dry, then crushed to a size of less than 75 μm and placed in the instrument. The relevant parameters were adjusted (the diffraction angle 2θ scan range was set to 5° to 90°, and the scan speed was set to 5°/min) before conducting the test. The micromorphology of the SMGM was investigated by using a Phenom pure scanning electron microscope (Phenom-World, Eindhoven, The Netherlands). Samples were dried in an oven at 105 °C. They were adhered to the observation platform using conductive adhesive, and surface dust was blown off using an ear syringe before being placed in the instrument for testing. The pore characteristics of the SMGM were examined by a nano Voxel 2740 high-resolution X-ray digital core computed tomography (Sanying Precision Instruments Co., Ltd., Tianjiin, China). The sample size was φ50 mm × 50 mm. Samples were placed on the CT bearing platform, relevant parameters were adjusted, and scanning was started. After scanning, visualization software was used for three-dimensional reconstruction and analysis of the pore structure.

### 2.5. Performance Requirements

The purpose of synchronous grouting in shield tunneling is to prevent segment uplift, reduce surface settlement, and enhance the overall stability of the tunnel [33,34]. Therefore, the grouting slurry should have good conveyance performance, filling performance, and suitable strength. This study focuses on the synchronous grouting in loess strata. Through laboratory tests, the compressive strength of the undisturbed soil was found to be 0.11 MPa. By consulting specifications, the literature, and on-site construction conditions, the performance requirements for the grouting material used in this project are as follows: 3 h flowability greater than 160 mm, setting time between 10 and 24 h, bleeding ratio less than 5%, volume shrinkage rate less than 5%, and compressive strengths of not less than 0.15 MPa at 3 d and 1 MPa at 28 d.

## 3. Results and Discussion

### 3.1. Fresh Properties

#### 3.1.1. Influence of Factors on Bleeding Ratio

**Figure 4:**
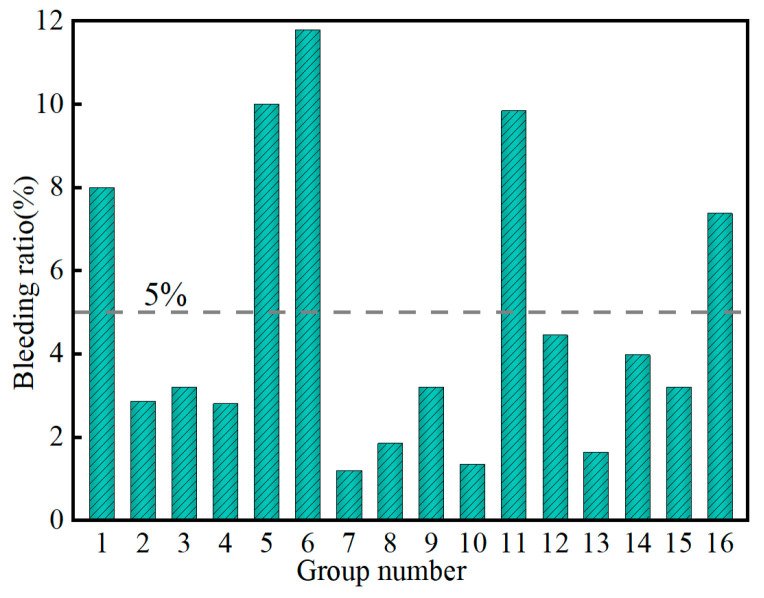
Variation in bleeding ratio of the SMGM.

**Figure 5:**
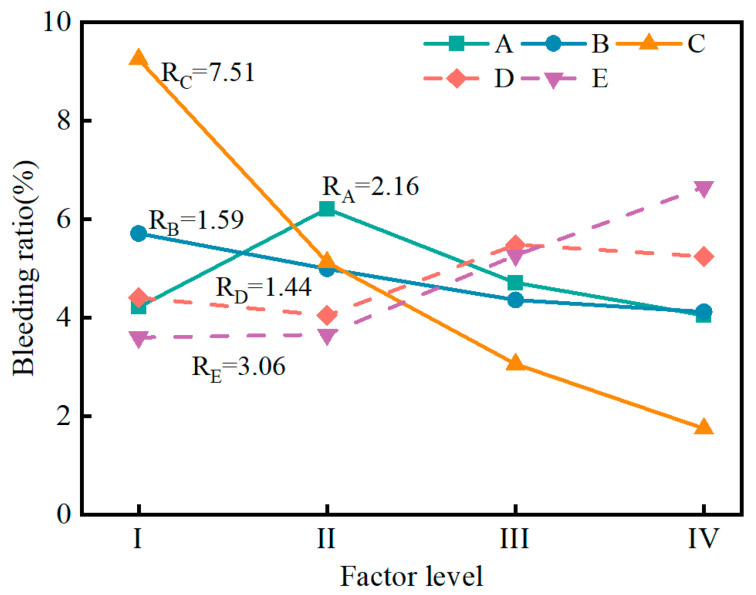
Range analysis of bleeding ratio.

Figure 4 shows the variation in the bleeding ratio of the SMGM. The bleeding ratio of the SMGM could be adjusted over a wide range (1.2~11.79%) by changing the mix ratio of grout, with NO. 7 having the lowest bleeding ratio (1.2%) and NO. 6 having the highest (11.79%). The bleeding ratios of NO. 2~NO. 4, NO. 7~NO. 10, and NO. 12~NO. 15 were below 5%. The influence of five factors on the bleeding ratio is shown in Figure 5. The range analysis result presented that the significance of each factor on the bleeding ratio was ranked in the order of R_C_ > R_E_ > R_A_ > R_B_ > R_D_. It showed that muck content had a great influence on the bleeding ratio of the SMGM (R_C_ = 7.51). The bleeding ratio of the SMGM decreased with the increase in the content of muck and fly ash. This is because clay minerals such as montmorillonite in the muck have a strong ability to adsorb water molecules [35]. As the muck increased, more water molecules were adsorbed by soil particles, the amount of free water molecules was reduced and thus the bleeding ratio was lowered. On the other hand, the bleeding ratio of the SMGM increased with the increase in the water–solid ratio. This is because as the water–solid ratio increased, more free water was not used for the hydration reaction, causing an increase in the bleeding ratio of the SMGM [36]. When the cement content was less than 8%, the bleeding ratio of the SMGM increased with the increase in cement content. However, when the cement content was greater than 8%, the bleeding ratio of the SMGM decreased with the increase in cement content. The admixture had the least impact on the bleeding ratio of the SMGM.

#### 3.1.2. Influence of Factors on Setting Time

**Figure 6:**
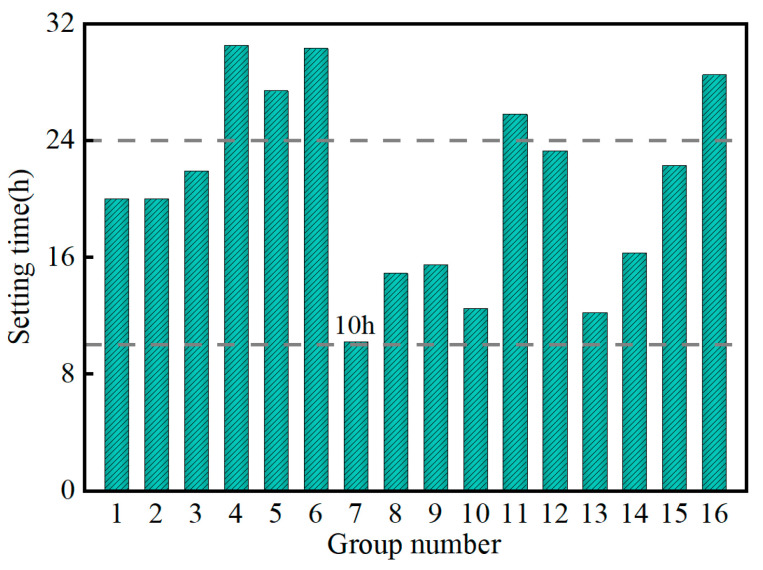
Variation in setting time of the SMGM.

**Figure 7:**
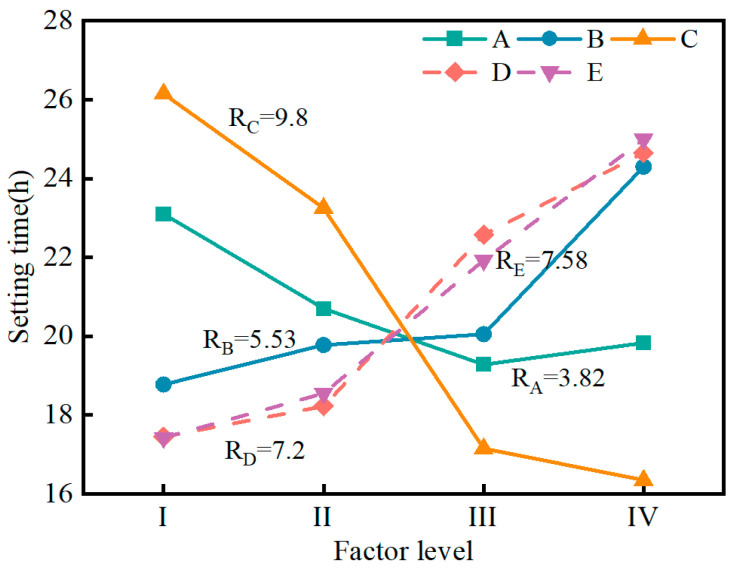
Range analysis of setting time.

Figure 6 shows the variation in the setting time of the SMGM. The setting time of the SMGM could be adjusted over a wide range (10.2~30.5 h) by changing the mix ratio of grout, with NO. 7 having the lowest setting time (10.2 h) and NO. 4 having the highest (30.5 h). The setting time of NO. 1~NO. 3, NO. 7~NO. 10 and NO. 12~NO. 15 was within 10~24 h. Figure 7 illustrates the influence of five factors on setting time. The range analysis result presented that the significance of each factor on the setting time was ranked in the order of R_C_ > R_E_ > R_B_ > R_A_ = R_D_. It showed that muck content and water–solid ratio had a great influence on the setting time of the SMGM (R_C_ = 9.8, R_E_ = 7.58). The setting time of the SMGM increased with the increase in the water–solid ratio, admixture content, and fly ash content. The admixture contains the retarder. It inhibits cement hydration. Large amounts of free water were filled inside the SMGM with the increase in the water–solid ratio, which made it difficult for the hydrated calcium silicate (C-S-H) gel formed by the cement hydration reaction to aggregate. Therefore, the setting time of the SMGM was prolonged. This was consistent with the results of G. Zheng’s study [37]. The setting time of the SMGM generally decreased with an increase in muck and cement content. This is because as the content of muck and cement increases, clay particles adsorb a large number of free water molecules [38], accelerating the hydration reaction of cement. Additionally, the negatively charged clay particles undergo very weak charge exchange with cement, aggregating into larger soil particles, which further shortens the setting time of the SMGM.

#### 3.1.3. Influence of Factors on Flowability

**Figure 8:**
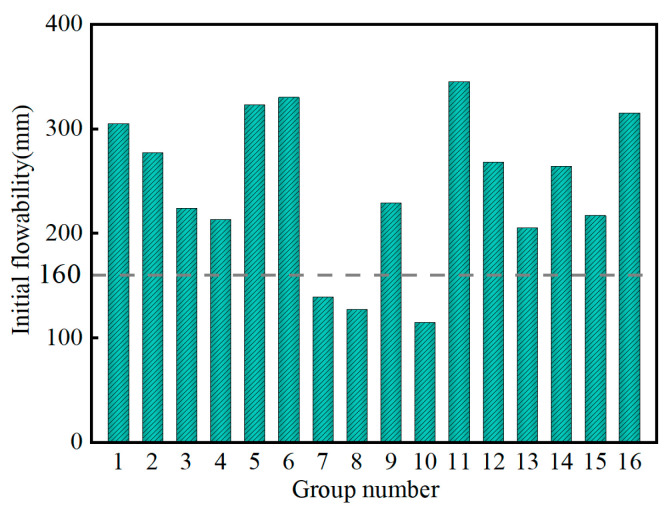
Variation in initial flowability of the SMGM.

**Figure 9:**
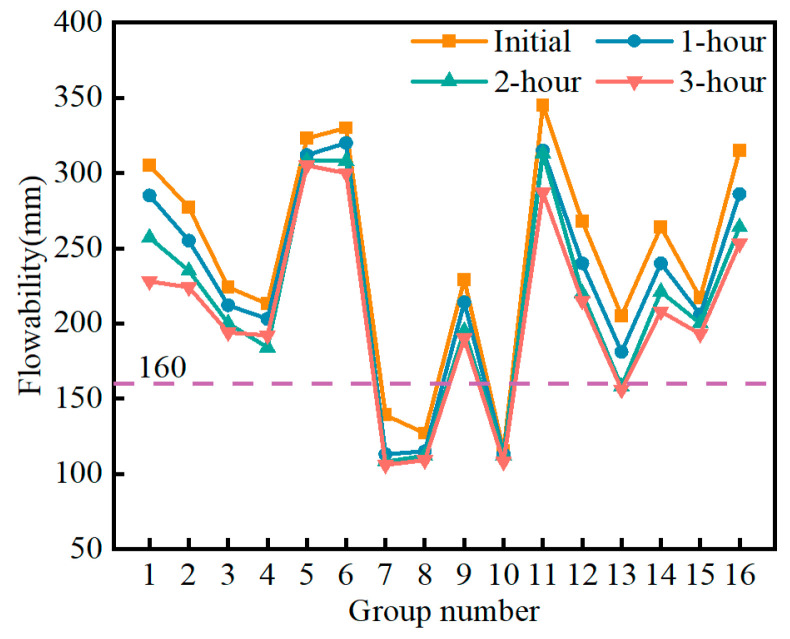
Variation in flowability of the SMGM.

Figure 8 shows the variation in the initial flowability of the SMGM. The initial flowability of the SMGM ranged from 115 mm to 345 mm, with NO. 10 having the lowest initial flowability (115 mm) and NO. 11 having the highest (345 mm). Figure 9 presents the flowability loss of the SMGM over time. The flowability of the SMGM decreased as time progressed because the number of free water molecules inside the slurry decreased and the C-S-H generated by cement hydration continuously aggregated, thereby gradually reducing the flowability of the SMGM. After 3 h from mixing, the flowability of NO. 1~NO. 6, NO. 9, NO. 11, NO. 12, and NO. 14~NO. 16 was greater than 160 mm.

**Figure 10:**
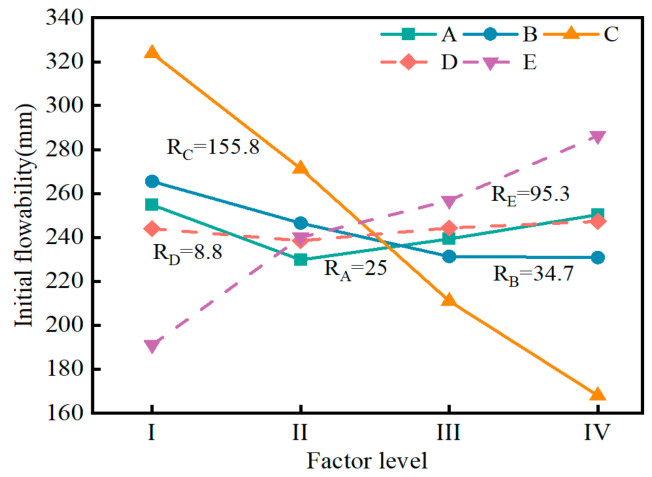
Range analysis of initial flowability.

Figure 10 illustrates the influence of five factors on flowability. The range analysis result presented that the significance of each factor on the flowability was ranked in the order of R_C_ > R_E_ > R_B_ > R_A_ > R_D_. R_C_ and R_E_ (R_C_ = 155.8, R_E_ = 95.3) were much higher than R_B_, R_A_, and R_D_, indicating that the flowability of the SMGM was mainly affected by muck content and water–solid ratio. The flowability of the SMGM decreased with the increase in muck content. This is because soil particles have a large specific surface area and a strong ability to adsorb water molecules, so an increase in muck content leads to a reduction in the number of free water molecules, thereby decreasing the flowability [38]. The flowability of the SMGM increased with the increase in the water–solid ratio because as the content of free water molecules rose, the friction between particles decreased, resulting in higher flowability of the slurry [27]. The flowability of the SMGM decreased with the increase in fly ash content. When the cement content was less than 8%, the flowability of the SMGM decreased with the increase in cement content. However, when the cement content was greater than 8%, the flowability of the SMGM increased with the increase in cement content. This may be because when the cement content is less than 8%, the increase in cement content leads to more hydration products within the slurry, causing it to become thicker and reducing its flowability. However, when the cement content exceeds 8%, the unhydrated cement particles act as lubricants, slightly increasing the flowability. The impact of admixture on the flowability of the SMGM was negligible.

### 3.2. Mechanical Properties

#### 3.2.1. Influence of Factors on Volume Shrinkage Rate

**Figure 11:**
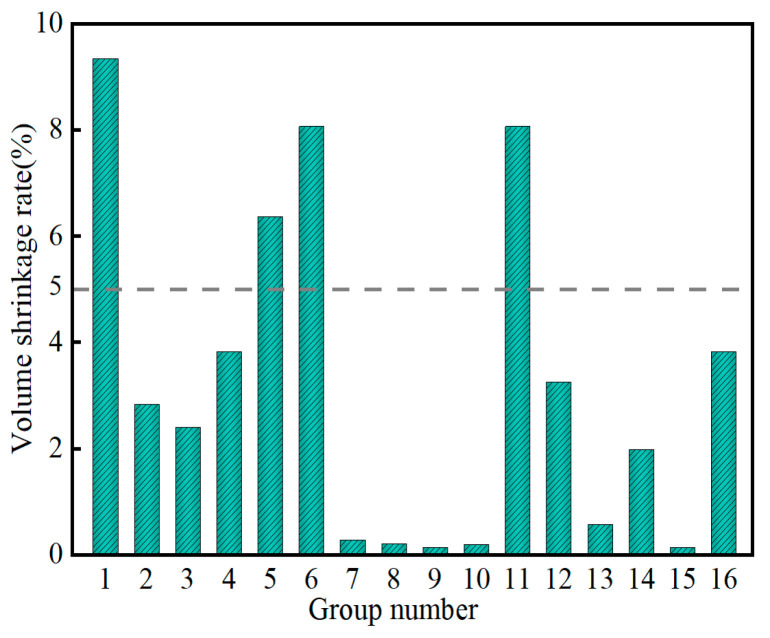
Variation in volume shrinkage rate.

**Figure 12:**
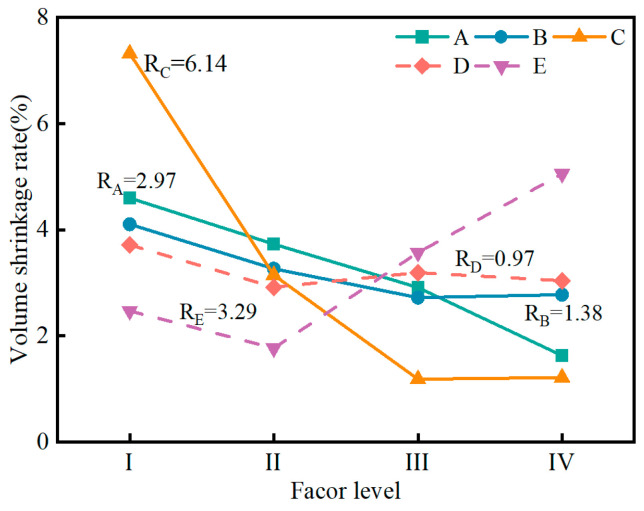
Range analysis of volume shrinkage rate.

Figure 11 shows the variation in the volume shrinkage rate of the SMGM. The volume shrinkage rate of the SMGM could be adjusted over a wide range (0.14~9.34%) by changing the mix ratio of grout, with NO. 9 and NO. 15 having the lowest volume shrinkage rate (0.14%) and NO.1 having the highest (9.34%). The volume shrinkage rates of NO. 2~NO. 4, NO. 7~NO. 10, and NO. 12~NO. 16 were below 5%. Figure 12 illustrates the influence of five factors on volume shrinkage rate. The range analysis result presented that the significance of each factor on the volume shrinkage rate was ranked in the order of R_C_ > R_E_ > R_A_ > R_B_ > R_D_. R_C_ (R_C_ = 6.14) was much higher than R_E_, R_B_, R_A_, and R_D_, indicating that the muck has a great influence on the volume shrinkage of the SMGM. The volume shrinkage rate of the SMGM decreased with the increase in the content of muck, cement, and fly ash. When the admixture content and water–solid ratio were less than 0.1 and 0.425, respectively, the volume shrinkage rate of the SMGM decreased with the increase in these two factors. However, when the admixture content and water–solid ratio exceed 0.1 and 0.425, respectively, the volume shrinkage rate of the SMGM increased with the increase in these two factors.

#### 3.2.2. Influence of Factors on Compressive Strength

**Figure 13:**
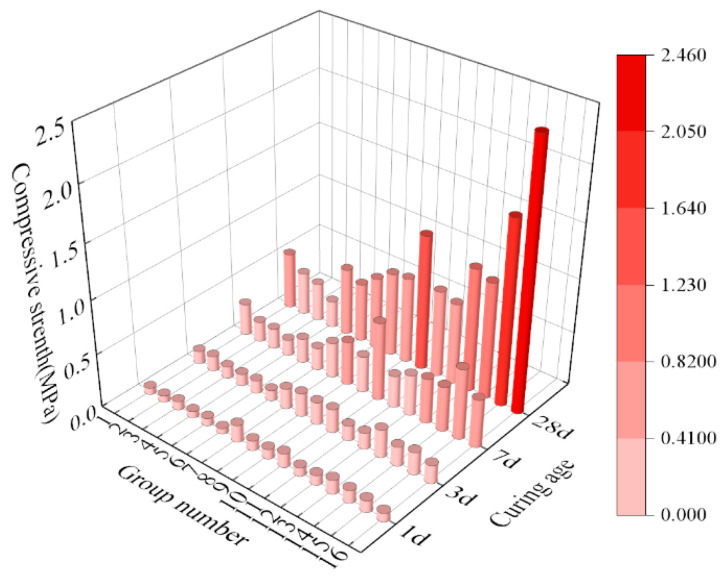
Compressive strength of the SMGM with different curing ages.

Figure 13 shows the variation in the compressive strength of the SMGM with curing age. The compressive strength of the SMGM at 1 d, 3 d, 7 d, and 28 d ranged from 0.05 to 0.16 MPa, 0.1 to 0.26 MPa, 0.15 to 0.73 MPa, and 0.25 to 2.46 MPa, respectively, indicating that the compressive strength of the SMGM gradually increased with the curing age [39]. At the same curing age (such as 28 d), the compressive strength of the SMGM increased with the cement content [40]. The compressive strength of NO. 10 and NO. 13~NO. 16 was greater than 0.15 MPa at 3 d and greater than 1 MPa at 28 d.

**Figure 14:**
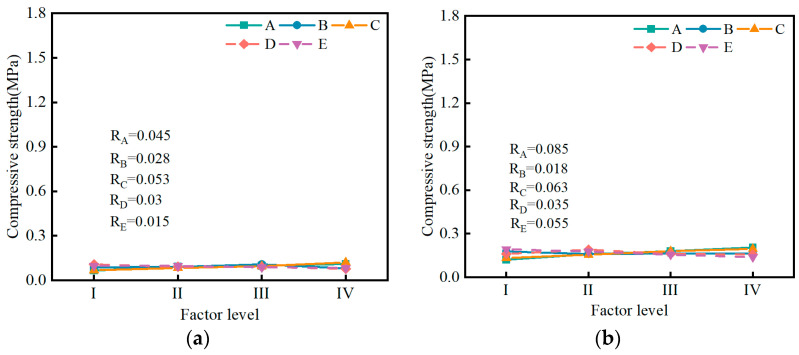

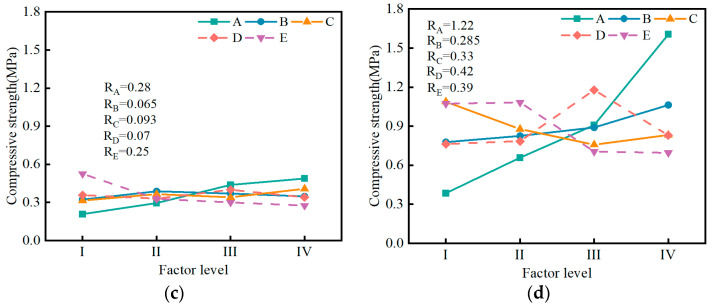
Range analysis of compressive strength of the SMGM at different curing ages: (**a**) 1 d curing age; (**b**) 3 d curing age; (**c**) 7 d curing age; (**d**) 28 d curing age.

Figure 14 illustrates the influence of five factors on the compressive strength of the SMGM at different curing ages. The range analysis result presented that the significance of each factor on the compressive strength was ranked in the order of R_A_ > R_E_ > R_C_ > R_D_ > R_B_. When the curing time was short, the range values of different factors were relatively small. As the curing time increased, the range value for cement content (R_A_ = 1.22) became much greater than those of the other factors, indicating that cement has a significant impact on the compressive strength of the SMGM. At 28 d of curing, the compressive strength of the SMGM increased with the increase in the content of cement and fly ash. This is because the hydration reaction of cement generates C-S-H gel and Ca(OH)_2_. In an alkaline environment, Ca^2+^ reacts with the active [SiO_4_]^4−^ and [AlO_4_]^5−^ in fly ash to form C-S-H. Additionally, Ca^2+^ undergoes ion exchange reactions with Na+ and K+ ions, causing changes in the electric double layer within the soil, leading to the attraction and aggregation of soil particles into larger particles. The aggregation of soil particles and the formation of C-S-H gel enhance the density of the grouted stone body, thereby increasing its compressive strength. The compressive strength of the SMGM decreased with the increase in the content of muck and the water–solid ratio [41,42,43]. When the admixture content was less than 0.2%, the compressive strength of the SMGM increased with the increase in admixture content. However, when the admixture content was greater than 0.2%, the compressive strength of the SMGM decreased with the increase in admixture content.

In order to improve the construction efficiency and ensure the effectiveness of grouting, this paper predicted the performance of the SMGM. Based on the results of 16 groups of orthogonal tests, the predictive regression model was established through multiple linear regression. The prediction model is shown in Table 5.

It can be seen from Table 5 that the fitted linear regression coefficients are all greater than 0.8, and the significance levels are all less than 0.05, indicating that the model is highly statistically significant. This can provide a reference for the practical application of the SMGM.

**Figure 15:**
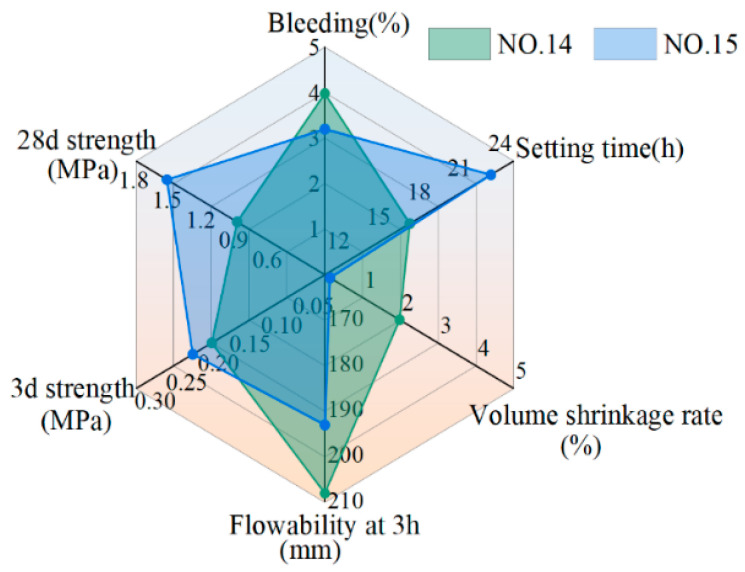
Radar graph of performance indexes of NO. 14 and NO. 15.

In summary, NO. 14 and NO. 15 meet the performance requirements of grouting slurry. The performance indicators of NO. 14 and NO. 15 are shown in Figure 15. To simplify the construction process (since NO. 15 requires additional admixture), NO. 14 can be selected as the mix proportion for synchronous grouting.

### 3.3. Microstructure Properties

To explore the internal structure of the SMGM and analyze the intrinsic relationship between their strength and microstructure, XRD, SEM, and CT tests were conducted on NO. 4 and NO. 14 samples cured for 28 d.

#### 3.3.1. XRD Analysis

**Figure 16:**
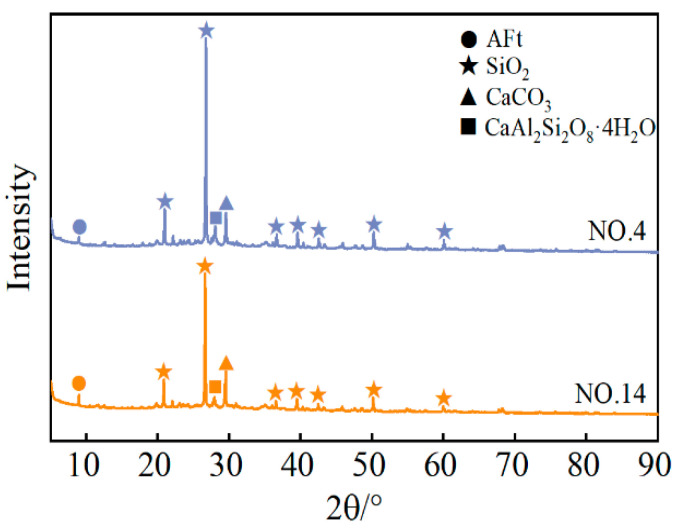
XRD results of NO. 4 and NO. 14 at 28 d.

XRD is a rapid method for detecting the phase composition of materials. The height of the diffraction peaks reflects the content of the phase components. The higher the peak, the greater the content. The XRD diffraction results of the SMGM are shown in Figure 16. The hardened SMGM contained SiO_2_ and CaAl_2_Si_2_O_8_·4H_2_O, originating from unreacted solid particles; AFt from the hydration products of cement; and CaCO_3_ from the chemical reaction between carbonates in the soil and the hydration product Ca(OH)_2_. The cement hydration reaction generated C-S-H, AFt, and Ca(OH)_2_ [44,45]. Part of the Ca(OH)_2_ reacted with carbonates in the soil to form CaCO_3_, while another part underwent pozzolanic reactions with SiO_2_ and Al_2_O_3_ in FA [46]. Compared with NO. 4, NO. 14 has a higher cement content, resulting in more hydration products and greater consumption of SiO_2_ in subsequent reactions. Therefore, NO. 14 exhibits lower SiO_2_ diffraction peak values and higher AFt and CaCO_3_ diffraction peak values than NO. 4.

#### 3.3.2. SEM Analysis

**Figure 17:**
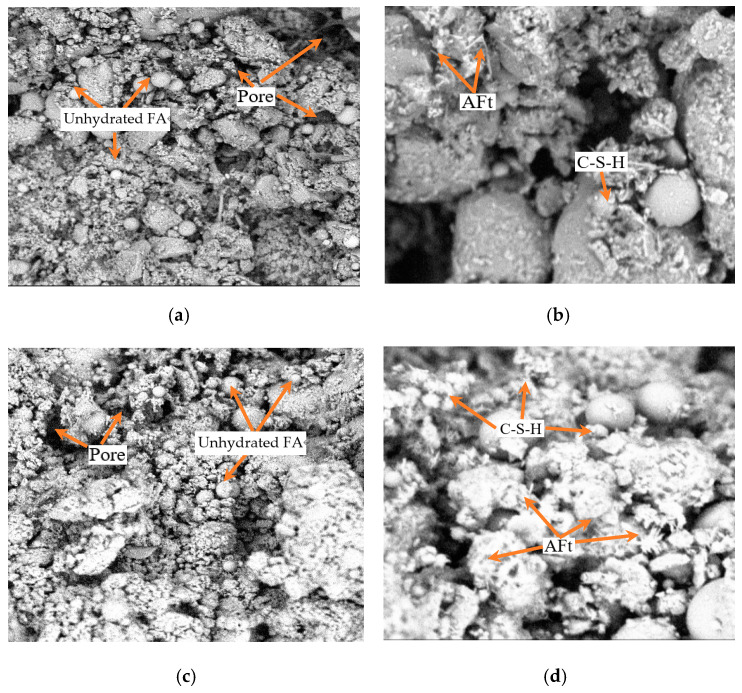
SEM analysis of NO. 4 and NO. 14. (**a**) The image of NO. 4 magnified 2000 times. (**b**) The image of NO.4 magnified 8000 times. (**c**) The image of NO. 14 magnified 2000 times. (**d**) The image of NO. 14 magnified 8000 times.

Figure 17 shows the micromorphology of NO. 4 and NO. 14 at 2 k and 8 k times. In NO. 4, there were various shapes and sizes of particles with relatively small contact areas between them, leading to a loose structure and the formation of numerous pores. Unhydrated FA was present as individual particles. In contrast, NO. 14 shows more aggregated soil particles, more gel substances, and smaller pores. This is due to the higher cement content in NO. 14, which generates more gel substances. These gel substances fill existing pores and bind the large particles aggregated through cohesion, reducing the porosity [47,48]. As a result, the internal structure of the SMGM becomes denser, significantly enhancing their mechanical properties.

#### 3.3.3. CT Analysis

**Figure 18:**
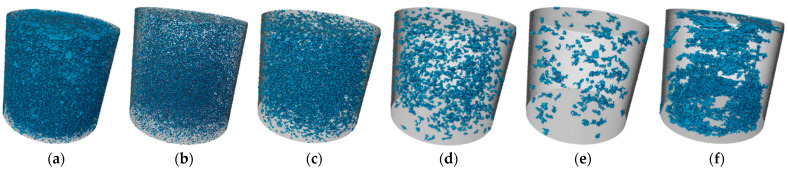
Three-dimensional distribution of NO. 4 macropores. (**a**) All equivalent diameters; (**b**) 28~200 μm equivalent diameter; (**c**) 200~400 μm equivalent diameter; (**d**) 400~600 μm equivalent diameter; (**e**) 600~800 μm equivalent diameter; (**f**) >800 μm equivalent diameter. Note: Blue is SMGM macropores and gray is the SMGM matrix under image resolution. The gray parts of the images also included SMGM pores, but not detectable at the image resolution.

**Figure 19:**
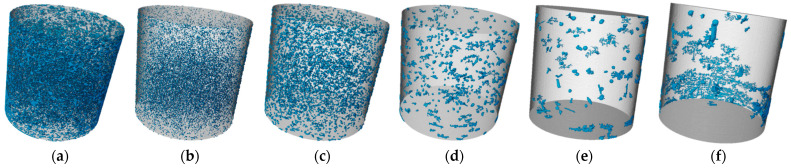
Three-dimensional distribution of NO. 4 macropores. (**a**) All equivalent diameters; (**b**) 28~200 μm equivalent diameter; (**c**) 200~400 μm equivalent diameter; (**d**) 400~600 μm equivalent diameter; (**e**) 600~800 μm equivalent diameter; (**f**) >800 μm equivalent diameter. Note: Blue is SMGM macropores and gray is the SMGM matrix under image resolution. The gray parts of the images also included SMGM pores, but not detectable at the image resolution.

Soil pores are categorized into macropores and micropores. According to Luxmoore, pores smaller than 10 μm are defined as micropores, which have a minor effect on the strength of the SMGM [49]. Due to the limitation of image resolution, this study analyzes only the characteristics of macropores in SMGM based on the 3D reconstruction model. Specifically, the porosity and pore numbers of NO. 4 and NO. 14 were divided into zones, and the distribution of macropores within these zones in the specimens is shown in Figure 18 and Figure 19. The porosity and pore number for each zone are listed in Table 6. The pore numbers for NO. 4 and NO. 14 decreased with increasing pore diameter, but their porosity does not show a clear trend. Compared to NO. 4, the porosity and pore numbers for each zone in NO. 14 were smaller, with the total porosity and total pore zone of NO. 14 being 22.66% and 42.51% of NO. 4, respectively. The reason for this is the higher cement content in NO. 14, which generates more gel substances through the cement hydration reaction and the pozzolanic effect of fly ash. These gel substances fill the internal pores. Additionally, due to particle aggregation, small particles cluster together to form larger particles. Consequently, these gel substances and soil particle aggregation reduce the pore numbers and decrease porosity [50].

**Figure 20:**
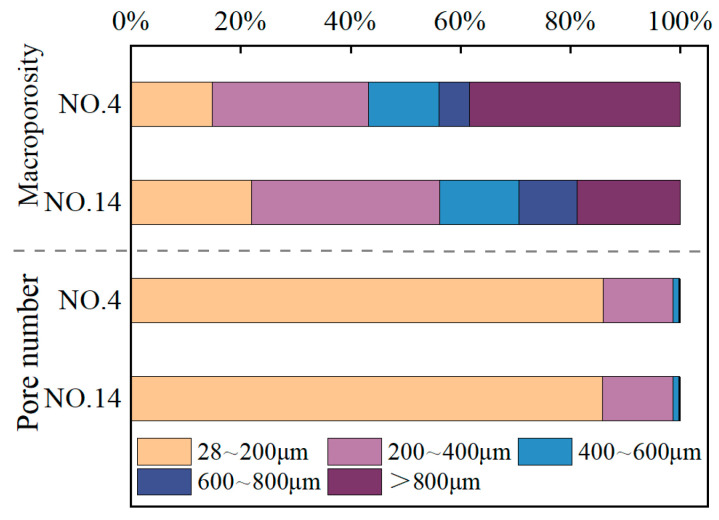
Pore proportion diagram of each zone.

The proportion of porosity and pore numbers for each zone of NO. 4 and NO. 14 within the macropores is shown in Figure 20. The macropores in NO. 4 and NO. 14 were mainly concentrated in the range of 28~200 μm. The pore numbers within the 28~200 μm range account for 86.04% and 85.92% of the total macropore numbers for NO. 4 and NO. 14, respectively. Compared to NO. 4, NO. 14 had an increased porosity in the 28~400 μm range and a significantly reduced porosity for pores larger than 800 μm.

In conclusion, compared to NO. 4, NO. 14 has a higher amount of hydration products. These hydration products and particle agglomeration significantly improve the internal pore structure of the SMGM, reducing their porosity [51,52]. Consequently, NO. 14 exhibits higher compressive strength, consistent with the results shown in Figure 13.

## 4. Conclusions

This study attempted to utilize shield muck for synchronous grouting. Through orthogonal experiments, the influence of the water–solid ratio and the content of cement, fly ash, muck, and admixture on the fresh properties, mechanical properties, and microstructure of the SMGM were investigated. The feasibility of shield muck as a synchronous grouting raw material was verified from both macro and micro perspectives. Based on the analysis of the experimental results, the following conclusions are drawn:

(1) The range analysis results indicate that the influence of muck content and water–solid ratio on the bleeding ratio, flowability, setting time, and volume shrinkage rate is greater than that of other factors. The influence of cement on the compressive strength is greater than other factors. A predictive model for the performance of the SMGM was established by using SPSS software (https://stats.oarc.ucla.edu/spss/, accessed on 14 August 2024), providing a reference for its practical application.
(2) Through the combination of macro and micro methods, it was found that a dense internal structure is the main reason for the higher compressive strength. The analysis suggests that the filling effect of the gel substances and the particle aggregation effect reduce the pores of the SMGM, significantly improving their pore structure and compactness.
(3) To meet the construction performance requirements and simplify the construction process, NO. 14 (12% for cement, 18% for fly ash, 50% for muck, 0.465 for water–solid ratio, 19.5% for river sand, 0.5% for bentonite) can be selected as the synchronous grouting for shield tunneling. Microstructural analysis results show that NO. 14 has a lower porosity and fewer pores compared to NO. 4.

## Figures and Tables

**Table 1:** Chemical compositions of materials (wt%).

Materials	SiO_2_	CaO	Al_2_O_3_	Fe_2_O_3_	MgO	K_2_O	SO_3_
Cement	22.06	41.96	11.49	3.06	1.67	1.06	2.14
Fly ash	40	10	30	4.2	2.5	1.1	2.42

**Table 2:** Properties of bentonite.

Material	Reading of Viscometer at 600 r/min/(mP·s)	Filter Loss/mL	75 μm Sieve Muck/%	Yield Point and Plastic Viscosity Ratio
Bentonite	35	12	2.1	2

**Table 3:** Orthogonal experimental design table.

Level	Factors				
	Cement Content (%) A	Fly Ash Content (%) B	Muck Content (%) C	Admixture Content (%) D	Water–Solid Ratio E
I	6	15	30	0	0.405
II	8	18	40	0.1	0.425
III	10	21	50	0.2	0.445
IV	12	24	60	0.3	0.465

**Table 4:** Orthogonal test design of the SMGM.

NO.	A (%)	B (%)	C (%)	D (%)	E	Bleeding Ratio (%)	Setting Time (h)	Initial Flowability (mm)	Volume Shrinkage Rate (%)	Compressive Strength (MPa)			
										1 d	3 d	7 d	28 d
1	6	15	30	0	0.405	8	20	305	9.34	0.06	0.12	0.3	0.53
2	6	18	40	0.1	0.425	2.86	20	277	2.83	0.06	0.14	0.19	0.4
3	6	21	50	0.2	0.445	3.2	21.9	224	2.4	0.08	0.11	0.19	0.36
4	6	24	60	0.3	0.465	2.8	30.5	213	3.82	0.06	0.11	0.15	0.25
5	8	15	40	0.2	0.465	10	27.4	323	6.36	0.07	0.12	0.23	0.63
6	8	18	30	0.3	0.445	11.79	30.3	330	8.06	0.05	0.1	0.21	0.55
7	8	21	60	0	0.425	1.2	10.2	139	0.28	0.16	0.19	0.33	0.67
8	8	24	50	0.1	0.405	1.85	14.9	127	0.21	0.09	0.22	0.41	0.78
9	10	15	50	0.3	0.425	3.2	15.5	229	0.14	0.09	0.21	0.34	0.8
10	10	18	60	0.2	0.405	1.35	12.5	115	0.19	0.13	0.22	0.65	1.26
11	10	21	30	0.1	0.465	9.84	25.8	345	8.06	0.08	0.14	0.3	0.81
12	10	24	40	0	0.445	4.45	23.3	268	3.25	0.09	0.15	0.38	0.76
13	12	15	60	0.1	0.445	1.64	12.2	205	0.57	0.13	0.26	0.47	1.15
14	12	18	50	0	0.465	3.98	16.3	264	1.98	0.12	0.18	0.42	1.09
15	12	21	40	0.3	0.405	3.2	22.3	217	0.14	0.11	0.21	0.66	1.72
16	12	24	30	0.2	0.425	7.38	28.5	315	3.82	0.08	0.17	0.45	2.46

**Table 5:** Performance prediction of the SMGM.

Variate	Expression	R^2^	*p*
F_b_	−1.47 − 5.413X_1_ − 20.275X_2_ − 25.193X_3_ + 395.5X_4_ + 49.313X_5_	0.871	<0.001
F_f_	−52.162 + 78.631X_1_ − 438.36X_2_ − 540.651X_3_ + 1550X_4_ + 1413.869X_5_	0.968	<0.001
F_s_	−33.837 − 67.998X_1_ + 52.213X_2_ − 33.934X_3_ + 2595X_4_ + 142.248X_5_	0.899	<0.001
F_v_	1.745 − 44.27X_1_ − 19.409X_2_ − 20.86X_3_ − 174.25X_4_ + 43.42X_5_	0.842	<0.001
F_3-c_	0.401 + 1.42X_1_ − 0.145X_2_ + 0.208X_3_ − 4.25X_4_ − 0.97X_5_	0.899	0.001
F_28-c_	1.962 + 18.952X_1_ + 2.658X_2_ − 0.805X_3_ + 59.5X_4_ − 6.952X_5_	0.816	0.002

Note: F_b_, F_f_, F_s_, F_v_, F_3-c_, and F_28-c_ were bleeding ratio, initial flowability, setting time, volume shrinkage rate, 3 d, and 28 d compressive strength, respectively. X_1_, X_2_, X_3_, and X_4_ represented the content of cement, fly ash, muck, and admixture, respectively. X_5_ represented the water–solid ratio.

**Table 6:** Macropore characteristic parameters of NO. 4 and NO. 14.

NO.	Total Porosity	28~200 μm	200~400 μm	400~600 μm	600~800 μm	>800 μm
4	7.06%	1.04%	2.01%	0.91%	0.39%	2.71%
14	1.6%	0.35%	0.55%	0.23%	0.17%	0.3%
NO.	total pore number	28~200 μm	200~400 μm	400~600 μm	600~800 μm	>800 μm
4	104,278	89,722	13,217	1101	155	83
14	44,330	38,088	5701	412	94	35

## Data Availability

The original contributions presented in the study are included in the article, further inquiries can be directed to the corresponding author.

## Abbreviations

SMGM	Shield muck grouting materials
XRD	X-ray diffraction
SEM	Scanning electron microscope
X-CT	X-ray computed tomography
FA	Fly ash
AFt	Ettringite
C-S-H	Hydrate calcium silicate
